# Clinical characteristics of the primary hepatic malignant fibrous histiocytoma in China: case report and review of the literature

**DOI:** 10.1186/1477-7819-10-2

**Published:** 2012-01-05

**Authors:** Dianbo Yao, Chaoliu Dai

**Affiliations:** 1 Department of Hepatobiliary and Splenic Surgery, Shengjing Hospital of China Medical University, Shenyang, Liaoning Province, China

**Keywords:** Malignant fibrous histiocytoma, Liver neoplasms, Diagnosis, Therapy

## Abstract

**Background:**

A malignant fibrous histiocytoma is a soft tissue tumor that most commonly occurs in the extremities, but rarely involves the liver. The clinical characteristics and therapeutic experiences of primary hepatic malignant fibrous histiocytoma are still limited.

**Methods:**

Two cases of primary hepatic malignant fibrous histiocytoma were analyzed retrospectively, and all the literature concerning primary hepatic malignant fibrous histiocytoma was analyzed.

**Results:**

In China, a total of 76 cases had been reported, among which 50 were men, with a male to female ratio of 1.9:1. Mean age of the patients was 51.0 years old, and more than 85 percent were older than 40 years. 82.9 percent (63/76) of hepatic MFH were solitary lesions, with tumor size ranging from 2.5 to 23.5 cm (average 10.3 cm). Major clinical presentation (78.4%) was abdominal pain or discomfort, accompanied with some other non-specific symptoms such as malaise, anorexia, weight loss, jaundice and fever, and small cases (14.9%) were asymptomatic. Computed tomography and ultrasound usually revealed the location of lesions. The rate of pre-operative misdiagnosis was extremely high, and 14.9 percent of patients were even misdiagnosed as a benign liver cyst, liver abscess or hematoma. Integrated resection was performed among the most cases (49/68), among which only a few ones (12 cases) were introduced to have no recurrence or metastasis or be still alive with no detail information provided, while among the cases with palliative operation or only a biopsy, the cases that were followed-up all died.

**Conclusions:**

Hepatic malignant fibrous histiocytoma is a rare malignant mesenchymal tumor. The variable features of clinical presentations and images make the diagnosis difficult. Though the prognosis of primary hepatic malignant fibrous histiocytoma was rather poor, integrated resection might provide a few cases a good opportunity for surviving, suggesting that surgery might be an effective treatment.

## Background

Since its initial description in 1964 by O'Brien and Stout[1], malignant fibrous histiocytoma (MFH) has been regarded as one of the most common soft tissue sarcomas of late adult life[2]. MFH primarily occurs in the extremities and less commonly involves the retroperitoneal spaces, abdominal cavity, or other sites [3]. The primary hepatic MFH is extremely rare, and there is still an incomplete understanding of the clinicopathology of primary hepatic MFH. In this study, two cases of primary hepatic MFH treated in our hospital are reported, and the literature of Chinese cases before June 2011 was reviewed and analyzed, to provide a better understanding of the clinicopathologic characteristics of this rare entity with an emphasis on its clinical characteristics and therapeutic experiences.

## Materials and methods

Clinical records of two patients with primary hepatic MFH treated from 2006 to June 2011 at the Shengjing Hospital of China Medical University were retrospectively reviewed. Surgeries were carried out in both patients, and the diagnosis was pathologically confirmed. Both of the patients were males, and their ages were 56 and 62 years old respectively. Routine preoperative examinations (physical examination, blood tests, ultrasonography, CT and/or MRI) and relevant tumor marker tests (A-fetoprotein, carcinoembryonic antigen and/or CA 19.9) were performed. One patient was followed up, while the other was lost.

China Academic Journal Full-text Database (CNKI), Chinese Scientific Journals Database (VIP) and Wan fang Database were used for searching Chinese reports about hepatic MFH before June 2011. Articles from the same institution were carefully studied to avoid the repetitive adoption of the saved material, and 50 articles including 91 cases were found.

A database including age, gender, symptoms, location, size of the tumor, metastasis or invasion of adjacent tissues, treatment and prognosis was created with all patients' characteristics. Every case that had at least six of these eight elements was characterized as well-documented. All of the non well-documented cases were excluded. 42 articles including 74 well-documented cases of primary hepatic MFH were left. Among the 74 cases, 3 cases were excluded because they were diagnosed as myxoid MFH which was no longer a subtype of MFH according to the 2002 WHO classification [4].

In addition, in the English literature, a total of 45 reported cases of primary hepatic MFH were found [5-31] (Table 1). Among the 45 cases, 8 cases were from China, and the related information of 5 cases in these 8 cases was also introduced in the Chinese literature. So finally, including 2 cases in our hospital, 76 cases of primary hepatic MFH in China were included in this review and analysis.

**Table 1:** Clinical Features of 45 Cases of Primary Hepatic Malignant Fibrous Histiocytoma in the English literature

References/Published date		Authors	No. Cases	Age/Sex	Presentation	Location	Size (Largest Dimension, cm)	Therapy	Follow-up/Outcome
5	1985	Alberti-Flor et al	1	59/M	Abdominal pain, anorexia, low fever, weight loss	Right and left lobe	18	Surgery	2 w/died of tumor
6	1985	Conran et al	1	61/M	Abdominal pain, anorexia, jaundice, weight loss	Right and left	7	Puncture biopsy	18 d/died of tumor
7	1986	Fukayama et al	1	38/F	Hepatic mass	Left lobe	7	Surgery	4 y/alive without recurrence
8	1987	Arends et al	1	78/F	Abdominal discomfort, anorexia,shortness of breath;	Right and left lobe	> 10	Palliative	6 d/died of tumor
9	1988	Bruneton et al	2	52/F	Abdominal pain	Right lobe	10	Surgery	2 y/alive without recurrence
				34/M	Abdominal pain	Right lobe	13	Palliative operation	6 mo/died of tumor
10	1988	Honda et al	1	71/F	Weight loss, fatigue, fever	Right lobe	> 10	Puncture biopsy, chemoembolization	4 mo/died of tumor
11	1988	Katsuda et al	1	61/M	Abdominal discomfort	Right lobe	8.5	Surgery and chemotherapy	6 mo/died of tumor
12	1991	Hamasaki et al	1	35/M	Abdominal fullness, palpable mass	Left lobe	9.1	Surgery, Intraarterial therapy, later liver transplant	34 mo/died of lung metastasis
13	1992	Akifuji et al	1	79/M	Anorexia, fatigue	Left lobe	8	Chemoembolization, Surgery	5 mo/recurrence
14	1992	Zornig et al	1	36/F	Abdominal pain	N/A	7	Surgery, postoperate chemotherapy	63 mo/alive without tumor
15	1992	McGrady et al	1	53/F	Left-sided chest pain	Left lobe	14 (operation)	Surgery, 5 years later recured, surgery again	9 y/alive without tumor after second surgery
16	1993	Reed et al	1	52/M	Spike fever	Right lobe	> 10	Surgery, postoperate chemotherapy	2 mo/died of metastasis
17	1994	Pinson et al	1	41/M	Abdominal pain, weight loss	Right and left	11.5	Surgery	10 and a half mo/died of recurrence and metastasis
18	1998	Fujita et al	1	70/M	Malaise, anorexia, fever, weight loss	Right and left	12	Puncture biopsy, Surgery	3 mo/died of tumor
19	1998	Wunderbaldinger et al	1	56/F	Palpable mass, lower extremity swelling	Right lobe	> 10	Surgery	N/A
20^†^	1998	Ferrozzi et al	3	62/F^†^	Abdominal pain, malaise, low-grade fever, and weight loss	Right lobe	> 12	Surgery	3 y/alive without recurrence
				67/F	Abdominal pain, weight loss, malaise and palpable mass.	Right and left	> 12	Biopsy, palliative chemotherapy.	N/A
				69/M	Abdominal pain, weight loss, fatigue and anorexia.	Right and left	> 12	Puncture biopsy, palliative chemotherapy	N/A
21^†^	1999	Yu et al	5	40-69(53)/4M, 1F	Abdominal discomfort*4, right shoulder and chest pain*1	Right lobe *3, left lobe *2	7-19 (mean 13)	Surgery*5	N/A*5
22	1999	Maekawa et al	1	68/M	Malaise	Right lobe	6	Surgery	N/A
23	2005	Anagnostopoulos et al	1	87/F	Abdominal pain, weight loss, low fever	Right lobe	12(CT)	Puncture biopsy	6 mo/died of tumor
24	2006	Ding et al	1	50/M	Abdominal pain, fatigue, weight loss	Right and left	14.2(CT)	Biopsy after laparotomy	2 mo/died of tumor
25^‡^	2007	Ye et al	1	50/M	Abdominal pain, weight loss	Left lobe	16	Surgery	4 mo/died of recurrence and metastasis
26	2007	Chen et al	1	70/M	Abdominal pain, weight loss	Right lobe	12.4(CT)	Palliative operation	1 mo/died of tumor
27^†^	2008	Li et al	7	77/F	Spiking fever, weight loss	Right lobe	10	Lobectomy	1 y/died of tumor
				54/M	Abdominal pain, weight loss	Left lobe	5.5	Lobectomy	4 y/died of tumor
				34/F	Abdominal pain	Right lobe	14	Liver and lung Lobectomy	4 mo/died of tumor
				80/F	Abdominal discomfort, weight loss	Right lobe	20	Palliative	1 y/died of tumor
				68/F	Abdominal pain	Right and left	2	N/A	N/A
				46/F	Abdominal pain	Right lobe	12	Gleevecineffective	3 mo/alive, tumor larger but no metastasis
				70/M^†^	Incidental finding	Right lobe	11	Lobectomy	4 y/no recurrence
28	2009	Sugitani et al	2	45/F	Abdominal pain	Left lobe	11	Surgery, 2 years and 5 months later recured, surgery again	34 mo/died of tumor metastasis
				70/F	Jaundice	Right lobe	12	Preoperate chemotherapy, Surgery	8 mo/died of tumor metastasis
29	2009	Kim et al	1	60/M	Abdominal pain, weight loss	Right lobe	14	Surgery	41 mo/alive without recurrence
30	2010	Caldeira et al	1	63/M	Abdominal discomfort, lower extremities edema and weight loss.	Right lobe	N/A	Puncture biopsy	45 d/died of tumor
31	2011	Cong et al	5	50-71(59)/2M, 3F	Abdominal pain or discomfort*4, weight loss*1	Right lobe *2, left lobe *1, right and left*1, N/A*1	2.5-16	Surgery*5	N/A*5

† indicates containing cases that were diagnosed as myxoid MFH; ‡ giant cell-type MFH

## Results

### Characteristic Features of Our Study Patients

#### Case 1

**Figure 1:**
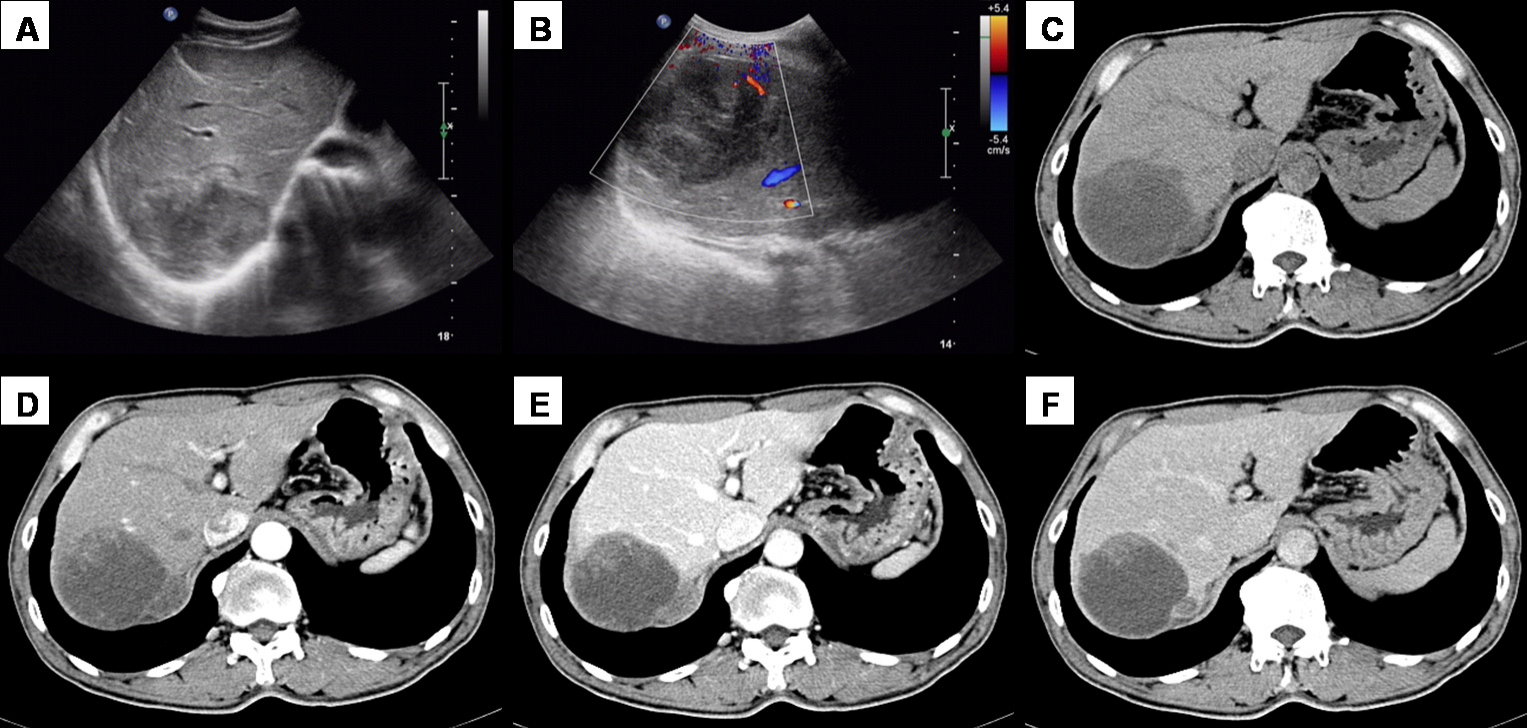
**A 56-year-old man with abdominal discomfort**. (A, B) Abdominal sonography revealed a hypoechoic mass with low vascularity. (C) Non-enhancement CT showed a heterogeneous hypodense mass with areas of cystic changes in the right lobe. (D- F) After the contrast injection, the mass was slightly enhanced, especially on the edge.

A 56-year-old man presented with discomfort in the right upper abdominal quadrant, nausea, without weight loss. Physical examination revealed a soft, non-tender abdomen. Ultrasonography (Figure 1A, B) revealed a hypoechoic mass with unmarked arrangement in the right posterior lobe. The echogenicity was inhomogeneous, and color Doppler study showed no pulsative flow signals, suggesting low vascularity in the mass. Non-enhancement CT (Figure 1C) showed a heterogeneous mass with areas of cystic changes in the right lobe. The size of the lesion was 6.8 × 6.6 cm, and the lesion was ill demarcated with the surrounding hepatic parenchyma. On contrast enhancement CT (Figure 1D-F), solid components showed slight enhancement, while the cystic components were never enhanced. Laboratory data showed that the liver function tests were all within normal ranges. HBsAg and HBcAb were positive. Levels of alpha-fetoprotein (AFP), carcinoembryonic antigen (CEA) and carbohydrate antigen 19-9 (CA 19-9) in serum were all within normal limits. Ultrasound-guided needle biopsy was performed, but the histopathological findings were only necrotic tissues. Pre-operative diagnosis was hepatic neoplasm, and local resection of the right lobe was performed. The surgical specimen measured 8 cm, and the mass was mostly solid, brown and partially white in colour, with focal necrotic and hemorrhagic foci. The pathological diagnosis was primary hepatic pleomorphic storiform MFH. Immunohistochemically, the tumor cells showed positive for vimentin, while stains for CK, EMA, Calponin, CD34, AFP, hepatocyte and S-100 were all negative. The patient has been followed up for 6 months to date, and is still alive, without a recurrence.

#### Case 2

**Figure 2:**
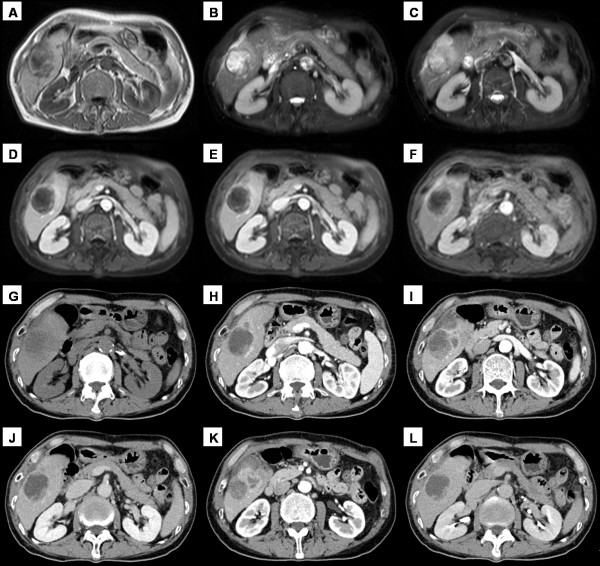
**A 63-year-old man with abdominal pain**. (A-C) MR images revealed a mass with low signal intensity on T1-weighted imaging and high signal intensity on T2-weighted imaging. (D-F) The periphery of the mass was slightly enhanced during the arterial, portal or delayed phase of contrast enhancement. (G) On CT, a heterogeneous hypodense mass was revealed. (H-L) After contrast material injection, the mass showed gradual, inhomogeneous enhancement, with decreased enhancement on delayed scans.

A 63-year-old man presented with intermittent twinge pain in the right upper abdominal quadrant for 10 days, without radiating pain, fever or weight loss. Physical examination on admission revealed that abdomen is flat and soft, and tenderness was in right upper abdomen, without rebound tenderness. Liver and spleen was untouched. MRI examination (Figure 2A-F) revealed a mass that was ill delineated from surrounding liver parenchyma, and the main signal intensity of the mass was hypo-intense compared with the liver, similar to that of skeletal muscle on T1-weighted imaging. The main signal intensity of the mass on T2-weighted images was higher than that in the liver, renal cortex or spleen. At subsequent CT (Figure 2G-L), a heterogeneous multi-nodular mass was confirmed, occupying the segment 6. The mass was slightly enhanced during the arterial phase of contrast enhancement, and the enhancement was decreased during the portal and delayed phase. Emission Computed Tomography (ECT) suggested that there was no formation of bone metastasis. Laboratory data showed a white blood cell count of 11.9 × 109/L, hemoglobin level of 131 g/L and a platelets count of 179 × 109/L. The liver function tests were all within normal ranges. Viral Hepatitis Testing revealed that HBsAg and HBcAb were positive. Alpha-Fetoprotein (AFP) was 1111.93 g/l (RR, < 25 g/l), and carbohydrate antigenic determinant (CA 19-9) was 12.46 u/ml (RR, < 37 u/ml). Pre-operative diagnosis was hepatic neoplasm, and the patient then underwent local resection of the right lobe. The cut surface of the resected tumor showed a multi-nodule appearance with necrosis, and gray yellow in colour. The pathological diagnosis was primary pleomorphic storiform MFH. Immunohistochemically, the tumor cells showed positive for vimentin and CD68, while stains for CK, CK8/18, CD35, CD21, CD1a, S-100, HMB45, MelanA, SMA, LCA were all negative. The patient was lost to follow-up.

### Analysis of reports in China

#### Cases in China (including 2 patients in our series and 8 cases reported in the English literature)

Combined with the 2 patients in our series, 76 patients in China have been reported (Table 2). Among the 76 patients, 50 were men, with a male to female ratio of 1.9:1. The mean age of the 71 patients which have a clear age or mean age record was 51.0 years old (range, 10-77 years), and 88.1 percent (52/59) of the patients were older than 40 years.

**Table 2:** Clinicopathological characteristics of primary hepatic malignant fibrous histiocytoma

	Number of patients	Percentage (%)
Sex	76	
Male	50	65.8
Female	26	34.2
Age (years)	59	
Older than 40 years	52	88.1
Yonger than 40 years	7	11.9
Clinical presentation	74	
Abdominal pain or discomfort of differing degrees	58	78.4
Asymptomatic	11	14.9
Obstructive jaundice	3	4.1
Fever	17	22.8
Loss of body weight	13	17.6
Nausea and anorexia	8	10.8
Location	66	
Left lobe	24	36.4
Right lobe	36	54.5
Right and left lobe	6	9.1
Diameter (cm)	76	
Larger than 5 cm	66	86.8
Smaller than 5 cm	10	13.2

Most of hepatic MFH were large neoplasms, and only 13.2 percent (10/76) were smaller than 5 cm. The exact sizes of hepatic MFH were mentioned in 59 cases, including 19 cases with diameter descriptions in radiological investigations. The mean diameter was 10.3 cm (range, 2.5-23.5 cm).

Among the 76 cases, 82.9 percent (63 cases) of the tumors were described as solitary lesions, while only 17.1 percent of the tumors were composed of multiple lesions. The information of tumor localization was provided in 66 cases, including 10 cases with multiple tumor nodules. Among the 10 cases, the tumor nodules of 4 cases were found in both right and left lobes and the tumor nodules of the remaining 6 cases were confined to one lobe of the liver, 3 in the left and 3 in the right lobe. Among the 56 cases with a solitary lesion, tumors were often found in the right lobe (33 cases), and less frequently in the left lobe (21 cases), and the lesions of the remaining 2 cases (3.6%) were in the middle part of the liver.

For clinical manifestation of hepatic MFH, 74 cases provided details. The symptoms were usually non-specific. 58 patients (78.4%) complained of abdominal pain or discomfort of different degrees, accompanied with loss of body weight in 12 cases, obstructive jaundice in 1 case, nausea and anorexia in 8 cases, and fever in 15 cases, including 2 cases with the temperature more than 39 degree. Nearly 14.9 percent (11 cases) of the patients were asymptomatic, with the tumors found during routine physical examinations or unrelated conditions. In addition, 2 patients were admitted to hospital for obstructive jaundice and 2 patients were for fever. The last one patient was admitted for loss of body weight and the lesion was found by the radiological investigation on admission.

A variety of radiological investigations were performed for the evaluation, including ultrasonography (US, 46 cases) and computed tomography (CT, 63 cases) in the vast majority of the patients, and magnetic resonance imaging (MRI) in only 16 patients. The tumors were described on cross-sectional imaging as solid, cystic or heterogeneous (solid and cystic) masses. Among the US investigations of 46 cases, 34 cases provided details. The tumors appeared hypoechoic in 10 cases, hyperechoic in 10 cases, anechoic in 2 cases, and showed a mixed pattern that may include extensive necrotic areas in 8 cases. In addition, the tumors were described as cyst-like lesions in 2 cases. Among the remaining 2 cases, the lesions of one case were not detected and the other case had a lesion in each lobe of the liver, a cyst-like lesion in the left lobe and a hypoechoic lesion in the right. We have detail CT descriptions of 49 cases. The lesions that were not detected in the US investigation were not detected either in the CT investigation. Another case had one lesion in each lobe of the liver, a hypodense mass in the left lobe and a cyst-like lesion in the right. Among the remaining 47 cases, on plain CT scanning, MFH appeared as hypodense masses with necrotic areas of differing degrees in 40 cases, cyst-like masses in 4 cases, mixed masses in 3 cases. 45 cases provided details on the enhancement scanning. The solid components or the cystic walls of 41 cases were introduced to be variably enhanced, from slightly to markedly, while the masses of the remaining 4 cases showed no enhancement. Among the 16 cases with MRI examinations, 2 cases provided no information of T1-weighted MR images. The T1-weighted MR images of the remaining 14 cases all showed low signal-intensity masses. T2-weighted MR images of all the 16 cases showed high signal-intensity solid masses. 11 cases provided details on contrast-enhanced dynamic MR images. A gradual enhancement of internal components was present in 10 cases, and no obvious enhancement was showed in only 1 case.

For laboratory examinations, 5 cases provided no details. It showed that 77.8 percent (35/45) of liver function tests and 60.8 percent (31/51) of hepatitis investigations were normal in the patients. About the tumor marker, only a few of patients have slightly elevated tumor marker levels, such as serum alpha-fetoprotein (AFP, 7/68), carcinoembryonic antigen (CEA, 1/41), carbohydrate antigen 19-9 (CA19-9, 3/34). 5 cases have the description of carbohydrate antigen 125 (CA125) examinations and only 1 patient has an elevated level.

Despite the technological advances, pre-operative diagnosis of hepatic MFH is still difficult, and the rate of misdiagnosis is extremely high. We had information about preoperative diagnosis for 67 patients, none of which, including one case with puncture biopsy before operation in our series, got the exact diagnosis. 48 cases were diagnosed as hepatic neoplasms, 8 cases were only diagnosed as masses of liver. In addition, 10 cases (14.9%) were even misdiagnosed as benign lesions of the liver, including hemangioma in 3 cases, hepatophyma in 5 cases, hepatic hydatid cyst in 1 case and hepatic cyst in 1 case. The remaining 1 case was only diagnosed as calculi of intrahepatic duct, and the hepatic mass was not discovered until surgery.

Surgical procedure was the main treatment for hepatic MFH (Table 3). Among the 68 patients whose treatments were available, 49 cases (72.1%) had integrated resection, 4 cases (5.9%) had palliative operation (local resection), 7 cases had (10.3%) puncture biopsy, and the other 8 cases (11.8%) were found unresectable at laparotomy and underwent biopsy. In one case with integrated resection, a lesion in the lung was also detected synchronously with the one in the liver, and left pneumonectomy was performed 50 days after operation. The lesion of the lung was demonstrated as metastatic MFH and no adjunctive therapy was performed afterward. Among the remaining 48 vases with integrated resection, 3 cases received chemotherapy, 2 cases received radiation therapy, 1 case received hepatic artery and portal vein subcutaneous embedding alternate infusion chemotherapy after operation, 1 case received hepatic arterial infusion chemotherapy twice because of tumor recurrence 2 months after operation, and 1 case received lymphadenectomy and 125I radiation therapy because of lymph nodes metastasis 3 months after operation. Among the 4 cases with palliative operation, 1 case received intrahepatic dehydrated alcohol injection and hepatic arterial chemoembolization six times and regular antituberculosis treatment because misdiagnosised as intrahepatic nonspecific inflammatory granuloma, HCC or tuberculosis respectively before operation, and after operation superselective arterial chemoembolization and hepaticarteriography chemoembolization were also performed, another case received drainage fenestrated with laparoscope because misdiagnosised great cyst in the left lobe of liver before laparotomy, and received palliative operation because of intraperitoneal plant. Among the 7 cases with puncture biopsy, 1 case received percutaneous cryoablation combined with transarterial chemoembolization. Among the 8 cases with biopsy after laparotomy, 2 cases received chemotherapy.

**Table 3:** Treatment and prognosis of primary hepatic malignant fibrous histiocytoma

	Number of patients
Treatment	68
Integrated resection	49
Palliative operation (local resection)	4
Puncture biopsy	7
Biopsy after laparotomy	8
Follow-up	42^†^
Recurrence or metastasis within a year	10
Recurrence or metastasis over a year	2
No recurrence or metastasis within a year	5
No recurrence or metastasis over a year	2
Died	16^‡^
Alive	6

^† ^including one case that was lost after finding the lesion was increasing 4 month after operation. ‡ including 15 cases that died within a year and one case that died without the surviving time provided.

Most cases of primary hepatic MFH were the type of pleomorphic storiform MFH. One case in the Chinese literature was the type of inflammatory MFH, which has not been reported in the English literature. In the English literature, the only case that was diagnosed as giant cell MFH was also from China. For immunohistochemical investigations, only 28 cases provided details. Immunohistochemically, the tumor cells showed typically positive for Vimentin(27/27), CD68(13/13), and MAC387(2/2), and AAT(13/14), Lysozyme(11/12), ACT(10/12) showed positive in most cases. AFP(5/5), FVIII(2/2), CEA(4/4), EMA(9/9), CK8/18(7/7), Hep parl(4/4), CD10(3/3), HMB-45(7/7), Actin(5/5), Desmin(13/13) showed all negative, and CK(1/12), CD34(1/5), S-100(1/10), SMA(1/5) were expressed only in a few tumors.

To our knowledge, follow-up periods ranging from one month to 10 years were mentioned for 42 patients. The 8 cases with no treatment introduction have no follow-up either. Among the 49 cases with integrated resection, a postoperative follow-up was conducted in 33 patients. 10 cases of these 33 patients had recurrence or metastasis within a year, 2 cases had recurrence or metastasis over a year, 5 cases had no recurrence or metastasis within a year, 2 cases had no recurrence or metastasis over a year, 8 cases died within a year, and the remaining 6 cases were still alive with no detail information provided (the follow-up periods of these 6 cases was 1 month, 3 months, 23 months, 26 months, 3 years and 10 years respectively). Among the 4 cases with palliative operation, 2 cases were followed-up. 1 case died of multiple organ failure 2 months after operation, and the other was lost after finding the lesion was increasing 4 month after operation. Among the 7 cases with puncture biopsy, 2 cases were followed-up, and died 1 month and half of 1 month respectively after discharge from hospital. Among the 8 cases with biopsy by operation, 5 cases were followed-up. 4 cases died 1 month, 2 month, 3 month and half of 1 year respectively after discharge from hospital, and 1 case died without the surviving time provided.

## Discussion

Malignant fibrous histiocytoma (MFH) was first reported in 1964 by O'Brien and Stout[1] and has been considered to be one of the most common soft tissue sarcomas of late adult life[2]. Based on its morphological pleomorphism, five histologic subtypes of MFH were described by Enzinger and Weiss: pleomorphic storiform, myxoid, giant cell, inflammatory, and angiomatoid [2]. Now, according to the current WHO classification, myxoid MFH and angiomatoid MFH are no longer the subtypes of MFH, and the terms myxofibrosarcoma and angiomatoid fibrous histiocytoma are used for them. Myxofibrosarcoma has been reallocated from the fibrohistiocytic category to the myofibroblastic list and angiomatoid MFH is now classified under the category of tumors of uncertain differentiation [32].

MFH primarily occurs in the extremities and less commonly involves the retroperitoneal spaces, abdominal cavity, or other sites [3]. Primary hepatic MFH is exceedingly rare, especially in comparison with MFH of other sites, or with the more common primary malignancies of the liver [2,33]. Since its original description in 1985[5], to date, only 45 cases have been described in the English literature, including recent reports by Li et al. [27], Sugitani M et al. [28], Kim HS et al. [29], Caldeira A et al. [30] and Cong Z et al. [31]. Among the 45 cases, 8 cases were from China [24-26,31]. In the English literature, Li et al presented a series of 7 cases of primary hepatic MFH (including one case that was classified as myxoid type), which is the largest series to date. In our study, we review all well-documented reported cases of hepatic MFH in China before June 2011, and the largest series in the Chinese literature include 11 cases, among which one case was also classified as myxoid type. The cases of primary hepatic MFH were mostly the type of pleomorphic storiform. One case in the Chinese literature was the type of inflammatory MFH, which has not been reported in the English literature. In addition, in the English literature, the only case that was diagnosed as giant cell-type MFH was also from China.

For epidemiological characteristics of hepatic MFH, our study confirms previous reports on this neoplasm as a malignant entity, often with a large mass and predominantly affecting adult patients. However, there are also some differences in our study with previous ones. In the previous study in the English literature, patients were all older than 34, whereas in our study it is found that there are also 4 patients younger than 15 years old, including 1 boy and 3 girls, suggesting that the primary hepatic MFH could also occur among young persons. In addition, in a recent study of 34 cases, Li and colleagues showed that there was no sex predilection in the hepatic MFH[27], while in our study, 50 of 76 patients in China were men, with a male to female ratio of 1.9:1, suggesting that there might be a slight male predominance for the incidence of this rare malignant tumor. Certainly, these results still waits further confirmed in the future study.

The clinical presentation of primary hepatic MFH is nonspecific, including abdominal pain or discomfort, systemic symptoms of low-grade fever, malaise, anorexia, jaundice and weight loss, and nearly 14.9 percent (11 cases) of the patients in our study were asymptomatic. Spiking fever might also be a clinical presentation, such as 2 cases in the study of Li and colleagues [27], and 2 cases in our study. Consistent with previous studies, our study also confirms that radiological investigations of primary hepatic MFH are various and nonspecific. The lesions could be homogeneous solid [21], heterogeneous dense with areas of necrosis or nearly complete cystic changes [29] in the ultrasonography (US), CT or MRI examinations. After contrast administration, the solid components or the cystic walls mostly demonstrate variable enhancement. Delay enhancement can be observed and the intensity depends on the amount of fibrosis [21]. It's worth noting that though the lesions were usually detected and located by radiological investigations, specially, 1 case that was diagnosed as inflammatory MFH by biopsy was misdiagnosed only as calculi of intrahepatic duct before surgery, and the multiple hepatic masses in the left lobe of liver were not discovered until surgery. Laboratory examination also lack available and helpful indexes for preoperative diagnosis. 77.8 percent (35/45) of liver function tests and 60.8 percent (31/51) of hepatitis investigations were normal and the negative percentage of serum alpha-fetoprotein(AFP), carcinoembryonic antigen (CEA), CA19-9 and CA125 were all rather high, suggesting these tumor markers were not suitable predictors for this malignant hepatic tumor either.

Duo to the lack of the specification for the clinical presentations, images or tumor markers, the pre-operative diagnosis of hepatic MFH was rather difficult and the rate of pre-operative misdiagnosis was extremely high. In our study, none of the patients got the exact diagnosis. In addition, 10 cases (14.9%) were even misdiagnosed as benign lesions of the liver, including hemangioma, hepatophyma, hepatic hydatid cyst and hepatic cyst. The misdiagnosis may usually make the effective treatment delayed, or even lead to error treatments, especially for the hepatic MFH with cyst masses. 1 case in our study received drainage fenestrated with laparoscope because misdiagnosised as a great cyst in the left lobe of liver before laparotomy, and afterward the patient received only palliative operation because of intraperitoneal plant, which might be caused by the drainage.

Surgical resection with negative resection margins is the mainstay of treatment for primary MFH of the liver. Though the prognosis of hepatic MFH is poor, there are still some cases with good prognosis and one case in our study was even alive 10 years after resection of the lesion. In the study of Li et al, it was also showed that up to 33% of patients survived for more than 2 years [27]. So, early diagnosis and effective treatment might be an effective method. Furthermore, repeated surgery after recurrence might be also effective [15,28]. Besides, consistent with previous studies, our study also suggests that adjunctive therapies, such as chemotherapy chemoembolization or radiation therapy, are not effective treatment for this rare tumor. In addition, one thing to be noted is that the patients with incomplete surgery or biopsy seem to have a worse prognosis. In our study, among the cases with palliative operation or only a biopsy, 9 cases were followed-up and there were 7 cases that died within half a year after discharge from hospital, 1 case that died without the surviving time provided and 1 case that was lost after the lesion was found to increase 4 month after operation. Moreover, none of the 8 cases that survived more than 2 years in the English literature just received a biopsy [7,9,12,14,15,27-29].

## Conclusions

Although hepatic MFH is a rare malignant mesenchymal tumor, it should also be considered in the diagnosis of large liver lesions. The features of clinical presentations and images are variable and the pre-operative diagnosis is difficult. Surgical resection is the most effective means for treating this rare tumor. Though the prognosis is poor, complete surgery with negative resection margins might provide a few of cases a good opportunity for surviving. Surgery again may be also useful for a recurrent tumor.

## Consent

Written informed consent was obtained from the patient for publication of this Case report and any accompanying images. A copy of the written consent is available for review by the Editor-in-Chief of this journal.

## Abbreviations

**MFH**: Malignant fibrous histiocytoma.

## Competing interests

The authors declare that they have no competing interests.

## Authors' contributions

Yao DB participated in the design of the study, searched, studied and analyzed the data, and drafted the manuscript. Dai CL conceived of the study, participated in its design and helped to draft the manuscript. All authors read and approved the final manuscript.
